# Systematic Analysis of Reproductive Barrier Types and Strengths in Interspecific Hybridization Involving *Magnolia crassipes*

**DOI:** 10.3390/plants15030374

**Published:** 2026-01-25

**Authors:** Zhe Zhang, Yingbing Hu, Chenfei Huang, Minhuan Zhang, Xingliang Wu, Xiaoling Jin, Yan Huang

**Affiliations:** 1 College of Landscape Architecture, Central South University of Forestry and Technology, Changsha 410004, China; zzxy.la@gmail.com (Z.Z.);; 2 College of Arts, Nanning University, Nanning 530200, China

**Keywords:** magnolia crassipes, interspecific hybridization, reproductive barriers, pollen-stigma interaction, fruit set, seed abortion, backcross

## Abstract

*Magnolia crassipes* is a valuable species in *Magnolia* sect. *Michelia* known for its unique purple flowers, but interspecific reproductive barriers limit its use in breeding. Using *M. crassipes* as the maternal parent, we performed 13 pollination combinations (one selfed control and crosses with 12 taxa spanning five sections). We assessed reproductive processes from pollen–stigma interaction to seed and seedling performance, and verified hybrids using SSR markers. Reproductive barriers are strongly associated with phylogenetic distance, shifting from pollen-adhesion failure in crosses with donors from distant-section, to abnormal pollen-tube guidance in cross with *M. denudata*, and to fruit initiation in crosses with pollen donors from sect. *Michelia*. Among these *Michelia*-donor crosses, prezygotic barrier strength varied among combinations, as reflected by differences in stigma germination and ovule entry rates, which strongly influenced the potential for fruit set success. Postzygotic barriers further reduced reproductive success via seed abortion (peaking at 83.8%). However, all germinated hybrids exhibited normal early growth. Notably, backcrossing with the F1 hybrid *M.* ‘Danxia’ significantly improved reproductive compatibility (seed abortion rate 6.3% and germination rate 100%). This study clarifies the key barriers in *M. crassipes* hybridization and provides a basis and practical strategies for its genetic utilization.

## 1. Introduction

Magnoliaceae species are prized ornamental trees characterized by their large, vibrant, and fragrant flowers, as well as their elegant tree architecture. Consequently, these features render them highly valued for landscape design and urban greening [1]. The family comprises more than 300 species. Taxonomically, recent revisions have subsumed all genera except *Liriodendron* Linnaeus into a broadly defined genus *Magnolia* Linnaeus, which is further divided into 15 sections [2]. Of these subdivisions, sect. *Yulania* Spach dominates in horticultural applications, with more than a thousand ornamental cultivars. Conversely, sect. *Michelia* Linnaeus remains relatively underutilized in breeding programs. This group, however, possesses rich germplasm resources and strong potential for ornamental improvement.

Section *Michelia* comprises approximately 70 species, which are mainly distributed in tropical and subtropical Asia [3]. Of these, nearly 40 occur in China, the center of their distribution and origin [4]. These plants are evergreen trees or shrubs characterized by long flowering periods and abundant, strongly fragrant flowers, all of which confer high ornamental value. However, floral color diversity within this section is limited, with most species bearing white or pale-yellow flowers [5]. In this context, *Magnolia crassipes* Y.W. Law, a China-endemic species with purple-red flowers, represents a valuable genetic resource for broadening the floral color spectrum of sect. *Michelia*. Interspecific hybridization is a fundamental and widely used strategy in plant breeding programs [6], and serves as a primary approach for the genetic improvement of Magnoliaceae. By reshuffling parental genomes through recombination, hybridization generates diverse offspring that provide the raw material for selecting desirable traits [7].

However, species maintain their genetic integrity through reproductive isolation. Consequently, successful interspecific hybridization must overcome a series of reproductive barriers. These barriers are generally categorized as prezygotic or postzygotic, depending on whether they occur before or after fertilization [8,9,10]. Prezygotic barriers typically involve pollen–pistil incompatibility [11] and pistil-length mismatch [12]. Postzygotic barriers often manifest as hybrid seed abortion [13], immature fruit abscission [14], hybrid seedling lethality [15,16,17], hybrid weakness [18], and hybrid sterility [19].

To overcome these barriers, breeders have developed various approaches. These include pollination techniques such as mentor, delayed [20,21], or cut-style pollination [22], as well as laboratory interventions like embryo rescue [23], ploidy manipulation [24], and the use of bridging species [25]. Despite the availability of these techniques, their effective application necessitates a precise diagnosis of the specific incompatibility mechanisms. Reproductive barriers exhibit significant variation in both strength and type, even between closely related species [9]. Total reproductive isolation typically results from the cumulative effects of multiple pre- and postzygotic barriers, which contribute unequally to isolation [26,27]. Consequently, systematically characterizing the specific nature of these barriers is a prerequisite for selecting the most appropriate overcoming strategies to improve breeding efficiency.

Current research on the hybridization of *M. crassipes* has primarily focused on the success or failure of crosses rather than reproductive processes [28]. To address this gap, we conducted controlled pollinations using *M. crassipes* as the maternal parent. This involved one selfed control and crosses with 12 taxa. These pollen donors were specifically selected for their taxonomic diversity, ornamental trait complementarity, and adaptability to the climate of Hunan. This study aims to (1) identify the types of reproductive barriers present in hybrid combinations involving *M. crassipes*; (2) evaluate the intensity of these barriers; and (3) investigate their potential underlying causes. Overall, this study provides a theoretical basis and practical guidance for the genetic utilization of *M. crassipes*. Furthermore, it offers a valuable reference for overcoming reproductive barriers in Magnoliaceae breeding programs.

## 2. Results

### 2.1. Paternal Pollen Viability

**Figure 1:**
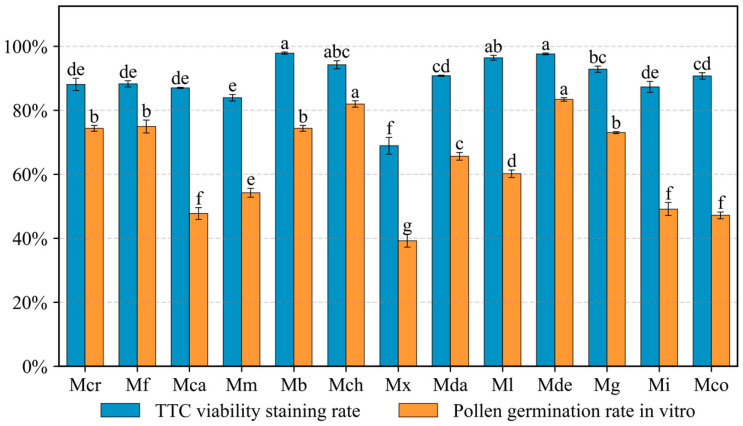
Pollen viability of the pollen donors. Blue bars represent pollen viability percentages determined by TTC staining, and orange bars represent pollen germination rates from in vitro assays. Data are presented as mean ± standard error (SE, *n* = 3). Different lowercase letters above the bars indicate statistically significant differences (*p* < 0.05) according to Duncan’s multiple range test. Abbreviations: Mcr, *M. crassipes*; Mf, *M. figo*; Mca, *M. cavaleriei* var. *platypetala*; Mm, *M. macclurei*; Mb, *M. balansae*; Mch, *M. chapensis*; Mx, *M.* ‘Xin’; Mda, *M.* ‘Danxia’; Ml, *M. lotungensis*; Mde, *M. denudata*; Mg, *M. grandiflora*; Mi, *M. insignis*; Mco, *M. conifera*.

2,3,5-Triphenyltetrazolium chloride (TTC) staining and in vitro germination assays demonstrated that pollen from all donors possessed sufficient viability for pollination (Figure 1). Although significant variations were observed among the pollen donors, even *M.* ‘Xin’, which had the lowest viability, maintained an in vitro germination rate of 39.3% (Table S1). Following the assessment of in vitro viability, the germination performance on the stigma was subsequently evaluated to observe pollen–pistil interactions.

### 2.2. Pollen Germination and Growth In Vivo

**Figure 2:**
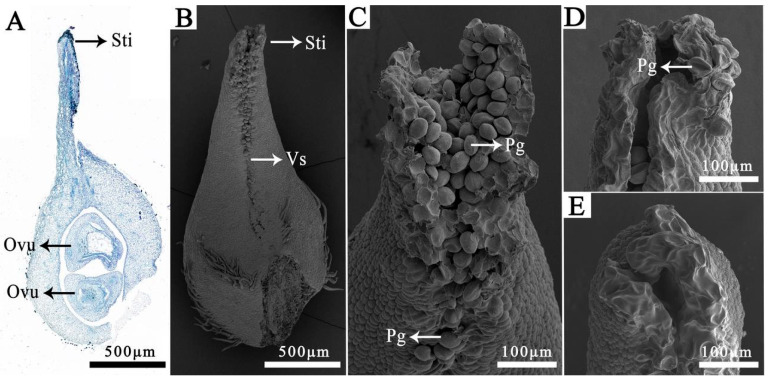
Carpel structure of *M. crassipes* and pollen adhesion after pollination. (**A**) Longitudinal section of a *M. crassipes* carpel. (**B**) SEM image of a single carpel. (**C**) Abundant adhesion of *M. chapensis* pollen on the stigma and ventral suture at 12 h after pollination (HAP). (**D**) Limited adhesion of *M. denudata* pollen on the stigma at 24 HAP. (**E**) No adhesion of *M. insignis* pollen on the stigma at 24 HAP. Abbreviations: Sti, stigma; Ovu, ovule; Vs, ventral suture; Pg, pollen grain.

The apocarpous gynoecium of *M. crassipes* typically consists of approximately 20 to more than 30 carpels. Each carpel bears two ovules arranged longitudinally along the ventral suture (Figure 2A). The stigma is located at the carpel apex, while the partially open ventral suture extends downward to the ovary (Figure 2B). Although pollen mainly adheres to the stigma, the ventral suture also exhibits stigmatic activity, supporting pollen attachment and germination (Figure 2C). SEM observations showed varying degrees of pollen adhesion: abundant adhesion was observed for pollen donors from sect. *Michelia*, whereas *M. denudata* (Desrousseaux) D. L. Fu (sect. *Yulania*) exhibited limited adhesion. In contrast, almost no pollen adhesion was detected for donors from sects. *Manglietia* Blume, *Magnolia*, or *Gynopodium* Dandy (Figure 2D,E), indicating a severe prezygotic barrier at the initial pollen–stigma recognition stage for these distant groups.

**Figure 3:**
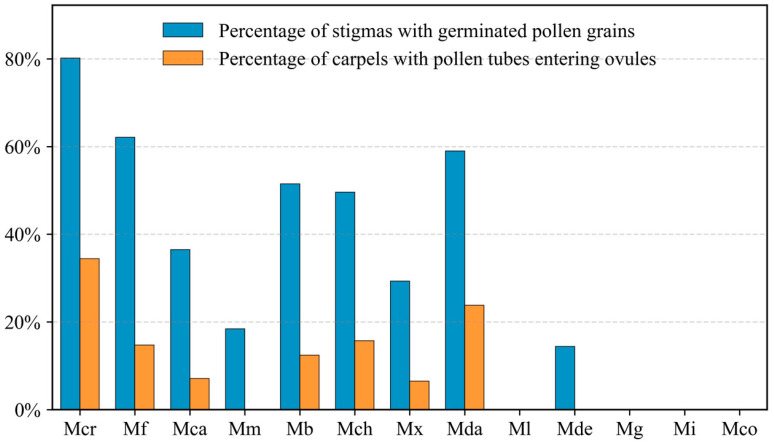
Quantitative assessment of in vivo pollen behavior of different combinations at 8 days after pollination (DAP). Blue bars represent the stigma germination rate (percentage of stigmas with germinated pollen grains), while orange bars indicate the ovule entry rate (percentage of carpels with pollen tubes entering the ovules). Abbreviations are consistent with Figure 1.

Pollen germination on the stigma and pollen tube growth within the carpels were examined by fluorescence microscopy. Figure 3 and Table S2 show the stigma germination rate (percentage of stigmas with germinated pollen grains) and ovule entry rate (percentage of carpels with pollen tubes entering the ovules) for each pollination combination.

**Figure 4:**
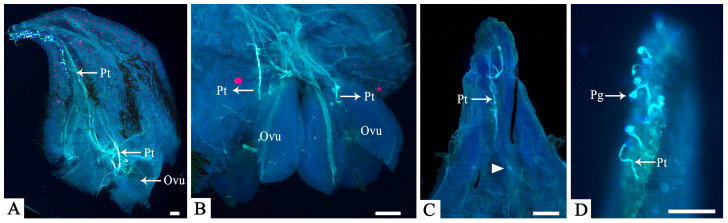
Fluorescence micrographs of in vivo pollen tube growth at 8 DAP. (**A**) Selfed control: Pollen tubes grew through the style and entered the ovules. (**B**) Cross with *M.* ‘Danxia’: Pollen tubes entered the ovules. (**C**) Cross with *M. macclurei*: Pollen tube growth was arrested within the style (indicated by the white triangle). (**D**) Cross with *M. denudata*: Pollen tubes exhibited abnormal coiling on the stigma surface. Abbreviations: Pt, pollen tube; Pg, pollen grain; Ovu, ovule. Scale bars = 200 μm.

Donors from sects. *Manglietia*, *Magnolia*, and *Gynopodium* exhibited in vitro pollen germination rates ranging from 49.1% to 73.0% (Table S1). However, no pollen germination was detected on *M. crassipes* stigmas, which corresponded to the absence of pollen adhesion observed earlier. In contrast, in vivo pollen germination occurred with all nine donors from sects. *Michelia* and *Yulania*, with *M. denudata* (sect. *Yulania*) showing the lowest stigma germination rate (14.4%) and pollen tubes that coiled on the stigma surface without penetrating the style (Figure 4D).

Among crosses with pollen donors from sect. *Michelia*, stigma germination rates varied widely (Table S2), ranging from 80.2% in the selfed control to 62.1% and 59.0% in crosses with *M. figo* (Lour.) DC. and *M.* ‘Danxia’, respectively, and dropping to 18.4% with *M. macclurei* (Dandy) Figlar. In the cross with *M. macclurei*, pollen tube growth was arrested within the style (Figure 4C), and no tubes were observed entering the ovules in the examined carpels. However, the development of two fruits in pollination trials suggests a rare occurrence of pollen tubes penetrating the ovule and achieving fertilization. Despite substantial variation in ovule entry rates, pollen tubes from the remaining combinations were observed entering the ovules in at least some carpels (Figure 4A,B). The highest ovule entry rate was recorded in the selfed control (34.4%), followed by the cross with *M.* ‘Danxia’ (23.8%). Conversely, the lowest rates were observed in crosses with *M. cavaleriei* var. *platypetala* (Handel-Mazzetti) N. H. Xia (7.1%) and *M.* ‘Xin’ (6.5%).

### 2.3. Fruit Set, Seed Number per Fruit, and Filled Seed Rate

**Figure 5:**
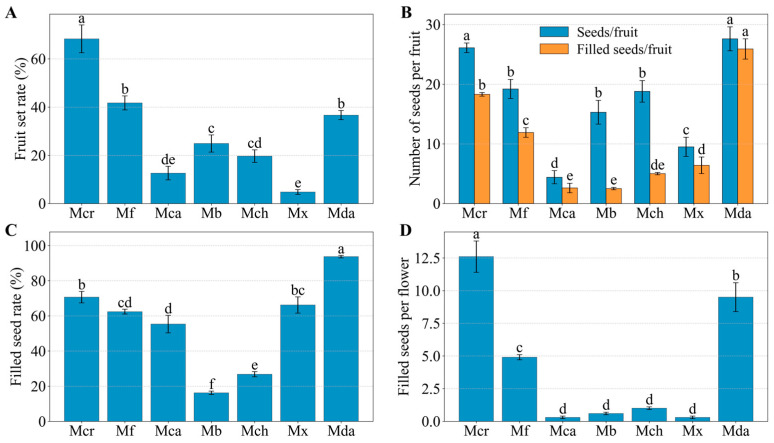
Fruit set, seed number per fruit, filled seed rate and filled seeds per pollinated flower of *M. crassipes* in crosses with different pollen donors. (**A**) Fruit set rate. (**B**) Comparison of total seed number and filled seed number per fruit. (**C**) Filled seed rate. (**D**) Filled seeds per pollinated flower, representing the integrated reproductive success per pollination event (Filled seeds per pollinated flower = Fruit set rate × Seed number per fruit × Filled seed rate). Data are presented as mean ± SE. Different lowercase letters indicate statistically significant differences (*p* < 0.05) based on Duncan’s multiple range test. Abbreviations correspond to those in Figure 1.

Fruit initiation was observed only when *M. crassipes* was crossed with pollen donors from sect. *Michelia*. Although two fruits were initially formed in the cross with *M. macclurei*, they abscised at an early developmental stage, and no mature fruits were obtained. Among the combinations where fruits reached maturity, significant differences were observed in fruit set rate, number of seeds per fruit, and filled seed rate (Figure 5; Table S3). The highest fruit set rate was recorded in the selfed control (68.3%), which was significantly higher than that of all hybrid combinations. Among the latter, crosses with *M. figo* and *M.* ‘Danxia’ were the most successful (41.7% and 36.7%, respectively), while the lowest rates were recorded for crosses with *M. cavaleriei* var. *platypetala* (12.6%) and *M.* ‘Xin’ (4.8%).

The number of total seeds per fruit ranged from 4.4 to 27.6 across all combinations (Figure 5; Table S3). The maximum values were recorded for the cross with *M.* ‘Danxia’ (27.6) and the selfed control (26.1), while the minimum values occurred in crosses with *M.* ‘Xin’ (9.5) and *M. cavaleriei* var. *platypetala* (4.4).

Filled seed rates among the successful combinations varied from 16.2% to 93.7% (Figure 5; Table S3). The maximum rate was recorded in the cross with *M.* ‘Danxia’ (93.7%), followed by the selfed control (70.6%) and the cross with *M.* ‘Xin’ (66.2%). In contrast, filled seed rates for crosses with *M. chapensis* (Dandy) Sima and *M. balansae* Aug. Candolle were 26.8% and 16.2%, respectively, corresponding to the highest seed abortion rates of 73.2% and 83.8% (Table S3).

The number of filled seeds per pollinated flower, representing the cumulative reproductive success, varied from 0.3 to 12.6 across the successful combinations (Figure 5; Table S3). The selfed control and the cross with *M.* ‘Danxia’ yielded 12.6 and 9.5 filled seeds per flower, respectively. A secondary peak was recorded for the cross with *M. figo* (4.9). For the remaining combinations, this metric was consistently lower, ranging from 0.3 (*M. cavaleriei* var. *platypetala* and *M.* ‘Xin’) to 1.0 (*M. chapensis*).

**Figure 6:**
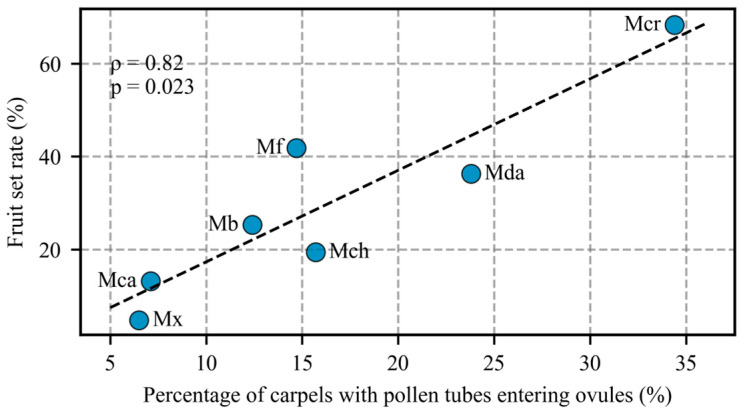
Correlation analysis between fruit set rate and ovule entry rate. The dashed line represents the linear regression trend. ρ indicates Spearman’s rank correlation coefficient, and *p* represents the statistical significance level. Abbreviations correspond to those in Figure 1.

We examined the relationship between fruit set rate and ovule entry rate across the combinations that yielded mature fruits (Figure 6). Spearman’s rank correlation analysis revealed a significant positive relationship between the two variables (ρ = 0.821, *p* = 0.023, *n* = 7).

### 2.4. Characteristics of Fruit and Seed Abortion

**Figure 7:**
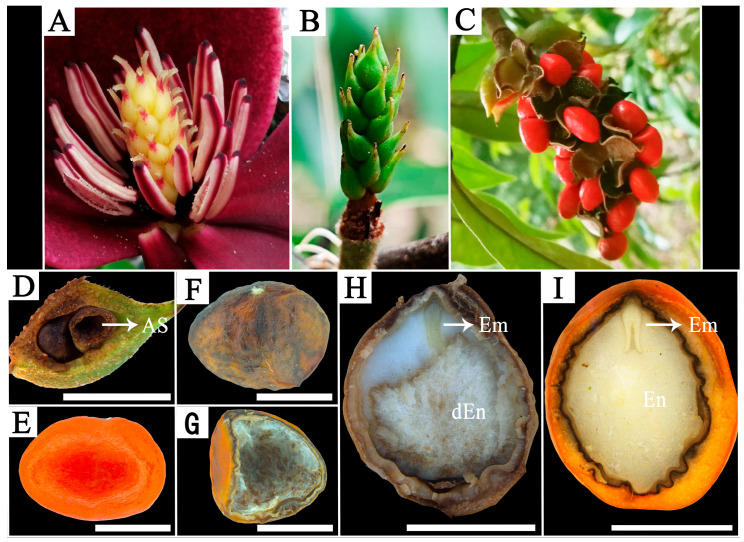
Morphological comparison of viable and aborted seeds and associated fruit development in *M. crassipes* crosses. (**A**) Gynoecium and androecium of *M. crassipes*. (**B**) Fertilized gynoecium at 30 DAP. (**C**) Mature aggregate fruit with dehisced follicles and exposed seeds. (**D**) Longitudinal section of a follicle from an abscised young fruit (*M. crassipes* × *M. macclurei*), showing aborted seeds (**E**) External view of a normal mature seed. (**F**,**G**) Aborted seed at fruit maturity: external morphology and dissection showing a hollow interior. (**H**) Longitudinal section of an aborting seed from the cross with *M. figo* at 130 DAP, showing partially degenerated endosperm (dEn) and an intact embryo (Em). (**I**) Longitudinal section of a normal mature seed, showing solid white endosperm (En) and a cotyledonary embryo (Em). Abbreviations: AS, aborted seed; dEn, degenerated endosperm; Em, embryo; En, endosperm. Bar = 5 mm.

Unfertilized gynoecia abscised within approximately 20–30 DAP. In contrast, fertilized gynoecia began to swell around 30 DAP (Figure 7B), with fruit size increasing markedly until approximately 70 DAP and remaining relatively stable thereafter. By around 175 DAP, the fruits were fully mature, and the follicles dehisced to reveal seeds enveloped in an orange-red sarcotesta (Figure 7C).

In the cross with *M. macclurei*, the only two fruits formed abscised prematurely between 50 and 55 DAP. Stereomicroscopic examination confirmed that all seeds within these fruits had aborted (Figure 7D). No young fruit abscission was observed in the other cross-combinations; however, seed abortion occurred during the later stages of development. Dissection of these seeds indicated that seed abortion was associated with endosperm degeneration. In seeds undergoing abortion, the distal portion of the endosperm was often necrotic, whereas both the embryo and its surrounding endosperm showed no obvious signs of deterioration (Figure 7E).

At fruit maturity, viable seeds were characterized by a smooth, orange-red sarcotesta containing solid white endosperm and a cotyledonary embryo (Figure 7F,G). In contrast, aborted seeds exhibited a shriveled sarcotesta with brown spotting and a hollow interior containing only flocculent or dried residues (Figure 7H,I).

### 2.5. Seed Weight, Germination, and Seedling Growth

**Figure 8:**
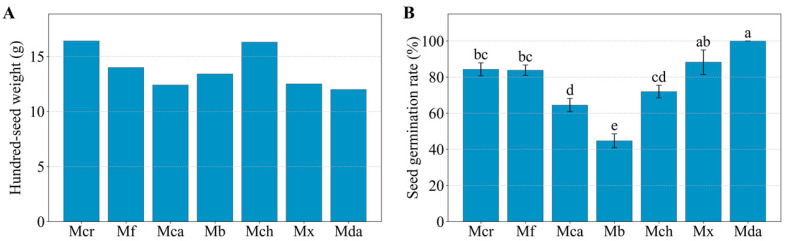
Hundred-seed weight and seed germination rate. (**A**) Hundred-seed weight (filled seeds). Note: For combinations with limited filled seed sets, the hundred-seed weight was calculated as (total weight/total seed count) × 100. (**B**) Seed germination rate. For (**B**), data are presented as mean ± SE (*n* = 3) and different lowercase letters indicate statistically significant differences (*p* < 0.05) according to Duncan’s multiple range test. Abbreviations correspond to those in Figure 1.

Hundred-seed weight among the successful combinations ranged from 12.0 g in the cross with *M.* ‘Danxia’ to 16.4 g in the selfed control, representing a 36.7% difference between the minimum and maximum values (Figure 8A; Table S4).

The variation in seed germination rate generally paralleled the pattern observed for filled seed rate. Germination rate were calculated based on filled seeds sown in the germination assay. Seeds from the cross with *M.* ‘Danxia’ exhibited the highest germination rate (100.0%), followed by those from crosses with *M.* ‘Xin’ (88.2%), selfed control (84.3%), and the *M. figo* cross (83.8%). The *M. balansae* cross yielded the lowest rate (44.7%) (Figure 8B). The cross with *M.* ‘Danxia’ produced seeds with the minimum hundred-seed weight (12.0 g) but the maximum germination rate (100.0%), showing no direct correspondence between seed weight and germination potential of the hybrid seeds.

Seedling survival and morphological traits were assessed at 130 days after sowing. A 100% survival rate was recorded for all combinations, with all seedlings exhibiting normal development and no signs of hybrid weakness, such as growth arrest, morphological deformities, or leaf chlorosis. At 130 days, seedling heights across the hybrid combinations ranged from 61.8 mm to 89.1 mm, compared to 70.1 mm in the selfed control (Table 1). All hybrids exceeded the height of the selfed control, with the exception of the cross with *M.* ‘Danxia’ (61.8 mm). Seedling heights differed significantly among crosses with different pollen donors. Although seedlings derived from the cross with *M.* ‘Danxia’ exhibited slower initial growth at 130 days, by one year they appeared taller than the selfed control based on visual observation.

### 2.6. Molecular Identification of Hybrids

To verify the hybridity of the putative hybrids, three seedlings from each of the six cross combinations were randomly selected for SSR marker analysis (Table 2). The polymorphic primers used in this assay yielded distinct banding patterns that differentiated the two parents. All F_1_ individuals from the six crosses carried the paternal alleles, indicating that every tested seedling was a true hybrid.

## 3. Discussion

### 3.1. Phylogenetic Distance Is a Key Factor Affecting the Type and Strength of Reproductive Isolation

Our study shows that, in *M. crassipes*-maternal crosses, the type and strength of reproductive isolation are strongly associated with phylogenetic distance. This result aligns with classical speciation theory, which predicts that reproductive isolation strengthens as genetic divergence accumulates [9,26]. According to recent phylogenetic data [2], the genetic distance between sect. *Michelia* and the sections to which the paternal parents belong decreases in the following order: sect. *Magnolia* > sect. *Manglietia* > sect. *Gynopodium* > sect. *Yulania*. Based on the timing and strength of the barriers, we categorized the crosses into three levels, reflecting a transition from complete prezygotic isolation to partial postzygotic isolation.

First, crosses with phylogenetically distant sections (sects. *Gynopodium*, *Manglietia*, and *Magnolia*) encountered the earliest and strongest barrier: pollen adhesion failure. In these crosses, we observed almost no pollen adhesion (Figure 2E) and no pollen germination was detected on the stigma. Pollen adhesion relies on specific recognition between pollen coat proteins and stigma receptors [29,30]. Studies in model plants show that this adhesion ability drops significantly as the evolutionary distance between the parents increases [31]. The failure of adhesion in our study suggests that the molecular recognition systems between *M. crassipes* and these distant species may have diverged, forming a strong prezygotic barrier.

Second, the barrier occurred at a later prezygotic stage in the cross with *M. denudata* (sect. *Yulania*). A limited number of pollen grains adhered and germinated on the *M. crassipes* stigma (at a rate of 14.4%), suggesting that basic recognition signals may be present. Nevertheless, these pollen tubes exhibited abnormal behaviors, such as curling and coiling on the stigma surface, and failed to enter the stylar transmitting tissue (Figure 4D), with an ovule entry rate of 0%. This is consistent with a potential disruption of the pollen tube guidance mechanism, which relies on precise molecular signaling between pollen and pistil [32,33,34].

Finally, crosses with pollen donors from sect. *Michelia* showed much weaker prezygotic barriers, with stigma germination rates ranging from 18.4% to 62.1% and ovule entry rates ranging from 0% to 23.8% among combinations. Although the observed ovule entry rates were low (or even undetected) in some combinations, these estimates are likely inherently probabilistic and influenced by limited sampling and microscopic detection constraints, among other factors. Importantly, all tested combinations in this section formed fruits, indicating that absolute prezygotic isolation does not exist in *M. crassipes* crosses with sect. *Michelia* pollen donors. However, postzygotic barriers, characterized by hybrid seed abortion, became prominent, and reproductive isolation therefore resulted from the cumulative contributions of both prezygotic and postzygotic barriers.

### 3.2. Prezygotic Barrier Strength Varies Among M. crassipes × sect. Michelia Crosses and Is Independent of Pollen-Donor Carpel Length

In our study, Spearman’s correlation analysis indicated a significant positive association between ovule entry rate and fruit set rate (ρ = 0.821, *p* = 0.023), although this analysis was based on a limited number of combinations (*n* = 7) and should therefore be interpreted cautiously. Similar positive relationships between ovule entry and fruit set have also been reported in other species [35,36,37]. Collectively, these findings suggest that ovule entry rate is a strong contributing factor to hybridization success.

Pistil-length mismatch is a significant prezygotic barrier reported in some eudicots such as *Nicotiana*, Rosaceae tribe Maleae, and *Prunus*. In those groups, pollen donors from species with longer pistils often achieve higher hybridization success, whereas “long pistil × short pistil” combinations are more likely to encounter reproductive barriers, as pollen tubes from short pistil species may lack the capacity to traverse the full length of a longer pistil [12,38,39]. However, in our study, we measured the carpel length of the pollen donors (Table S5) and found no significant correlation between the pollen donors’ carpel length and the ovule entry rate (Figure S1). For instance, crosses with *M. figo* and *M. chapensis* (which have short carpels) encountered only weak barriers, whereas those involving *M. macclurei* and *M.* ‘Xin’ (which have long carpels) exhibited strong incompatibility. This is likely due to the ancestral apocarpous morphology of Magnoliaceae. Unlike eudicots with fused styles, *M. crassipes* carpels have a partially open stigmatic surface extending along the ventral suture (Figure 2B,C). This arrangement may facilitate pollen grains to adhere and germinate along the suture, potentially reducing the distance pollen tubes must traverse within the carpel and alleviating physical constraints associated with total carpel length.

### 3.3. Seed Abortion Is the Critical Bottleneck in Postzygotic Barriers

Seed abortion is widely recognized as the major postzygotic barrier in plant hybridization [40], and our findings appear consistent with this view. For crosses with sect. *Michelia* pollen donors, seed abortion emerged as the key postzygotic barrier. The severity of abortion, measured as the abortion rate (100%—filled seed rate), varied sharply across combinations. The rates ranged from 6.3% in the cross with *M.* ‘Danxia’ to 73.2% and 83.8% in crosses with *M. chapensis* and *M. balansae*, respectively, and reached 100% (complete seed abortion) in the cross with *M. macclurei* (Table S3).

We identified two distinct morphological patterns of seed abortion in our study. First, early abortion occurred in the cross with *M. macclurei*, preceding young fruit drop. This phenomenon is potentially consistent with the role of developing seeds as a major source of auxin that sustains early fruit growth [14]. Accordingly, it is hypothesized that complete seed abortion may curtail seed-derived hormonal signals and thereby promote fruit abscission. Notably, in our other crosses, fruits matured successfully even when they contained only a single viable seed. Together, these observations suggest that fruit abscission in these combinations may not be an independent reproductive barrier but rather a secondary physiological consequence of complete early seed abortion. Second, late seed abortion associated with endosperm degeneration was observed in the other combinations (Figure 7). Dissection and stereomicroscopic observation revealed that in seeds undergoing late-stage abortion, necrosis was typically localized in the distal portion of the endosperm, while the embryo remained morphologically intact. Abnormal endosperm development is a common cause of hybrid seed failure [41], and in many species, it has often been linked to imbalanced expression of imprinted genes [42]. Imprinting patterns can evolve rapidly [43,44] and somewhat independently of phylogenetic distance [40]. This mechanism could potentially help explain the large variation in abortion rates observed in our study among combinations, even though all pollen donors belong to the same section (sect. *Michelia*).

Furthermore, our results suggest that seed weight may not be a reliable predictor of seed viability. Specifically, while the seeds from the cross with *M.* ‘Danxia’ were the lightest among all combinations, they achieved the highest germination rate (100%). Crucially, once germinated, all hybrid seedlings exhibited normal growth and showed no apparent signs of hybrid lethality or weakness. This suggests that postzygotic barriers in these combinations are largely confined to seed development. Consequently, once this critical developmental bottleneck is bypassed—for instance, through embryo rescue—there may be favorable prospects for obtaining vigorous hybrid progeny.

### 3.4. Restoring Cross-Compatibility Through Backcrossing: The Role of Bridge Parents

*M* ‘Danxia’ is an interspecific F_1_ hybrid derived from *M. crassipes* × *M. macclurei*. Therefore, using it as a pollen donor for *M. crassipes* represents a classic “(A × B) F_1_ × A” backcross. We found that this backcross exhibited remarkable reproductive compatibility. Its filled seed rate (93.7%) and seed germination rate (100%) significantly surpassed those of other hybrid combinations, and were even higher than the selfed control. This success stands in sharp contrast to the complete failure (100% early seed abortion) observed in the direct cross with *M. macclurei*. These findings suggest that backcrossing may help alleviate genetic incompatibilities, thereby providing a practical strategy to reduce severe seed abortion and effectively utilize genetic resources from parents that are otherwise difficult to hybridize directly.

## 4. Materials and Methods

### 4.1. Plant Materials and Study Site

**Figure 9:**
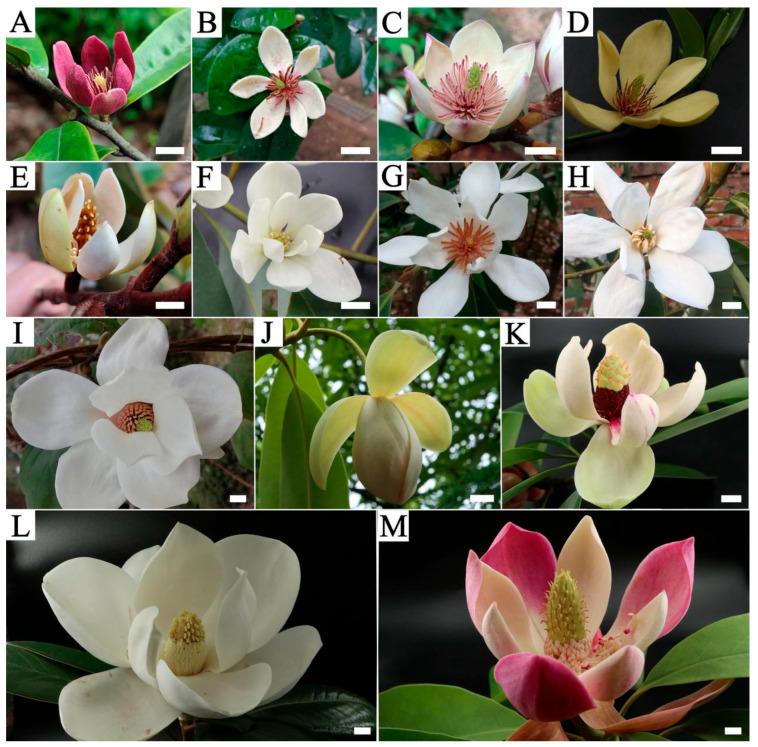
Floral morphology of the parental materials used in this study. (**A**) *Magnolia crassipes*; (**B**) *M. figo*; (**C**) *M.* ‘Danxia’; (**D**) *M. chapensis*; (**E**) *M. balansae*; (**F**) *M. macclurei*; (**G**) *M. cavaleriei* var. *platypetala*; (**H**) *M.* ‘Xin’; (**I**) *M. denudata*; (**J**) *M. conifera*; (**K**) *M. lotungensis*; (**L**) *M. grandiflora*; (**M**) *M. insignis*. Bar = 1 cm.

The study was conducted at the Magnolia Garden of the Hunan Forestry Science and Technology Demonstration Park (113°2′45.89″ E, 28°6′56.32″ N) in Changsha, Hunan Province, China. Thirty vigorous *M. crassipes* trees (>15 years old) cultivated in this garden were selected as maternal parents. Pollen was collected from pollen donors (Figure 9) located either in the same garden or on the campus of Central South University of Forestry and Technology (113°0′13.31″ E, 28°8′25.47″ N). All parental trees were healthy individuals maintained under routine horticultural management. Their ornamental traits are summarized in Table S6, while carpel length was recorded in Table S5.

### 4.2. Pollen Collection and Viability Assessment

Flowers were collected at the onset of anthesis prior to anther dehiscence. Stamens were detached and allowed to dehisce at room temperature. The collected pollen was then desiccated over silica gel and stored at −20 °C. Before pollination, viability was assessed using two methods: (1) TTC staining, involving incubation in 0.5% 2,3,5-triphenyltetrazolium chloride solution at 35 °C for 2 h; and (2) in vitro germination, using species-specific optimized liquid media (Table S7) at 25 °C in darkness for 6 h. Observations were performed using an Olympus BX53 microscope (Olympus, Tokyo, Japan). Three replicates were established for each species, with five randomly selected fields (>30 pollen grains per field) examined per replicate. Germination was defined as a pollen tube length exceeding the grain diameter.

### 4.3. Artificial Pollination

Artificial pollination was conducted in the spring of 2022. Thirteen combinations were established using *M. crassipes* as the maternal parent, comprising one selfed control and cross combinations with different pollen donors. The experiment followed a randomized design with three replicates per combination, and typically 60 flowers were pollinated per replicate. Operations were performed on sunny mornings (7:00–10:00). Flowers in the female stage with peak stigmatic receptivity were emasculated (retaining tepals), pollinated, and immediately bagged. Bags were removed 4 DAP, followed by periodic monitoring of gynoecium development.

### 4.4. Microscopic Observation of Pollen–Pistil Interactions

For each combination, ten flowers were harvested at 8 DAP, and gynoecia were fixed in FAA solution for 48 h. Subsequently, carpels were dissected and rehydrated through a graded ethanol series, followed by softing in 1 mol/L NaOH at 65 °C for 3 h. After staining with 0.1% water-soluble aniline blue for 24 h, squashed preparations were examined under a Leica DM i8 (Shinjuku City, Japan) inverted fluorescence microscope.

Gynoecia were harvested at 12 and 24 HAP for SEM observation of pollen adhesion on the stigmatic surface. Excised carpels were subjected to double fixation in glutaraldehyde and OsO_4_, followed by graded ethanol dehydration and substitution with isoamyl acetate. After critical point drying and sputter coating, specimens were imaged using an FEI Nova Nano SEM450 (Hillsboro, OR, USA) scanning electron microscope.

### 4.5. Fruit Set Rate, Seeds per Fruit, and Filled Seed Rate

Fruits were bagged prior to maturity and harvested at 175 DAP, characterized by follicle dehiscence and the exposure of orange-red sarcotesta. For each combination, the fruit set rate, total seed number per fruit, and number of filled seeds were recorded. Filled seeds were identified using a water flotation test, in which sinking seeds were classified as filled and floating seeds as aborted.

### 4.6. Seed Germination and Seedling Growth Assessment

After removal of the sarcotesta, hundred-seed weight was measured. Seeds were then stratified in moist sand at 6 °C for one month. Before sowing, only filled seeds (identified by the water flotation test in Section 4.5) were disinfected in 0.5% KMnO_4_ for 20 min and sown in plug trays with three replicates per combination. The substrate consisted of leaf mold, peat, and perlite (7:2:1, *v*/*v*/*v*), and seeds were covered with approximately 2 cm of soil. Seed germination and seedling growth were monitored regularly. At 130 days after sowing, germination rate, seedling survival rate, and plant height were recorded.

### 4.7. Hybrid Identification

When hybrid seedlings developed two true leaves, three individuals from each cross were randomly selected, and fresh young leaves were collected for DNA extraction using the DNAsecure Plant Genomic DNA Extraction Kit (DP320, Tiangen Biotech Co., Beijing, China). DNA purity was assessed by measuring the OD_60_/_280_ ratio with a NanoDrop 2000 spectrophotometer (Thermo Fisher Scientific, Waltham, MA, USA), and integrity was checked by 2.5% agarose gel electrophoresis. The forward primer of each SSR marker (Table S8) was labeled at the 5′ end with FAM fluorescence for genotyping. PCR amplification was performed in a 20 μL reaction mixture containing 10 μL of 2× PCR Mix, 1 μL of each primer, 3 μL of DNA template, and 5 μL of ddH_2_O. The cycling program consisted of an initial denaturation at 94 °C for 4 min, followed by 10 cycles of 94 °C for 35 s, 65 °C for 15 s, and 72 °C for 45 s, with the annealing temperature decreasing by 1 °C per cycle, then 35 additional cycles of 94 °C for 35 s, 55 °C for 35 s, and 72 °C for 35 s, and a final extension at 72 °C for 5 min. PCR products were mixed with LIZ500 size standard and formamide, denatured, and loaded with 1 μL product and 7 μL mixture per well. Fragment analysis was carried out using an ABI 3730 (Thermo Fisher Scientific, Waltham, MA, USA) capillary electrophoresis system, and genotyping was performed with GeneMarker software v2.6.3 (SoftGenetics, USA). Seedlings showing paternal-specific bands were identified as true hybrids.

### 4.8. Statistical Analysis

Statistical analyses were performed using SPSS 25.0 (IBM Corp., Armonk, NY, USA). One-way analysis of variance (ANOVA) followed by Duncan’s multiple range test was conducted to determine significant differences among treatments (*p* < 0.05). Spearman’s rank correlation was used to assess associations between selected variables. Data are expressed as mean ± standard error (SE). Figures were generated using Python 3.10, utilizing the Matplotlib 3.1.7 library for data visualization.

## 5. Conclusions

This study systematically characterizes the reproductive barriers limiting interspecific hybridization in *M. crassipes*. We found that the type and strength of these barriers appear to show a gradient based on phylogenetic distance. In crosses with distant sections (*Magnolia*, *Manglietia*, and *Gynopodium*), hybridization failed due to the absence of pollen adhesion, whereas the cross with *M. denudata* (sect. *Yulania*) proceeded beyond adhesion but displayed abnormal pollen-tube guidance. When pollen donors were from sect. *Michelia*, fruits formed, suggesting that prezygotic barriers were weaker or partially overcome in these crosses.

However, among crosses with sect. *Michelia* donors, prezygotic barrier strength varied among combinations and was associated with the observed variation in fruit set rate. Notably, their strength showed no significant correlation with the carpel length of the pollen donors. This differs from phenomena observed in some eudicots, suggesting that incompatibility may be primarily due to physiological pollen–pistil interactions, rather than to physical structural limitations.

Following fruit initiation, seed abortion proved to be the main postzygotic bottleneck. This varied significantly among different combinations, appearing as either early abortion accompanied by young fruit drop or late abortion after morphological formation which was associated with endosperm degeneration. However, those seeds that successfully germinated produced hybrid seedlings with normal growth. Moreover, backcrossing with an F_1_ hybrid significantly improved reproductive compatibility in our study, suggesting that backcrossing may help alleviate genetic incompatibilities and reduce severe seed abortion in otherwise difficult combinations.

In summary, this study identifies the key reproductive barriers in *M. crassipes* hybridization, providing a theoretical basis and practical implications for the utilization of genetic resources.

## Figures and Tables

**Table 1:** Plant height of progeny from different combinations at 130 days after sowing.

Combinations	Seedling Height (mm)
*Magnolia crassipes* × *M*. *crassipes* (Selfed control)	70.1 ± 1.2 c
*M*. *crassipes* × *M*. *figo*	75.1 ± 1.3 bc
*M*. *crassipes* × *M*. *cavaleriei* var. *platypetala*	78.9 ± 3.0 b
*M*. *crassipes* × *M*. *balansae*	76.6 ± 1.9 b
*M*. *crassipes* × *M*. *chapensis*	89.1 ± 1.9 a
*M*. *crassipes* × *M*. ‘Xin’	79.8 ± 1.9 b
*M*. *crassipes* × *M*. ‘Danxia’	61.8 ± 1.9 d

Data are presented as mean ± SE (*n* = 8) and different lowercase letters indicate statistically significant differences (*p* < 0.05) according to Duncan’s multiple range test.

**Table 2:** SSR marker analysis of the parents and their F_1_ progeny.

Cross Combination	SSR Locus	Maternal	Paternal	F_1_ Individual			Purity (%)
Mcr × Mf	SSR72	174/186	180/180	174/180	174/180	174/180	100
	M02	137/137	130/130	130/137	130/137	130/137	
Mcr × Mb	SSR72	174/186	176/184	174/184	174/184	184/186	100
	MMA72	223/223	229/240	223/229	223/229	223/229	
Mcr × Mx	M02	137/137	145/145	145/145	137/145	137/145	100
	MMA72	223/223	236/236	223/236	223/236	223/236	
Mcr × Mch	SSR82	289/289	285/285	285/289	285/289	285/289	100
	M29	236/245	255/259	245/255	245/259	245/255	
Mcr × Mca	M29	236/245	241/241	241/245	236/241	236/241	100
	SSR82	289/289	272/289	289/289	272/289	272/289	
Mcr × Mda	SSR82	289/289	285/291	289/291	285/289	285/289	100
	MMA72	223/223	211/211	211/223	211/223	211/223	

Abbreviations correspond to those in Figure 1.

## Data Availability

The data presented in this study are available on request from the corresponding author.

## Supplementary Materials

The following supporting information can be downloaded at: https://www.mdpi.com/article/10.3390/plants15030374/s1, Figure S1: Correlation analysis between carpel length and ovule entry rate; Table S1: Pollen TTC staining rate and in vitro germination rate of the 13 pollen donors; Table S2: Numbers of carpels observed for pollen germination on the stigma and pollen tube entry into ovules; Table S3: Fruit and seed set in *Magnolia crassipes* following pollination with different pollen donors; Table S4: Seed weight and germination performance of *Magnolia crassipes* following pollination with different pollen donors; Table S5: Carpel length of the pollen donors; Table S6: Taxonomy, role, and ornamental characteristics of the parental taxa used in this study; Table S7: Optimized medium components for in vitro pollen germination of the pollen donors; Table S8: Sequences of polymorphic SSR primers used for hybrid identification in this study.
