# Supplementary material for: Systematic Analysis of Reproductive Barrier Types and Strengths in Interspecific Hybridization Involving Magnolia crassipes
Source: Plants (Basel). 2026 Jan 25;15(3):374. doi: 10.3390/plants15030374 (PMC12899510; doi:10.3390/plants15030374)
Supplement: Supplementary file 1 [file plants-15-00374-s001.zip › Figure S1.pdf]

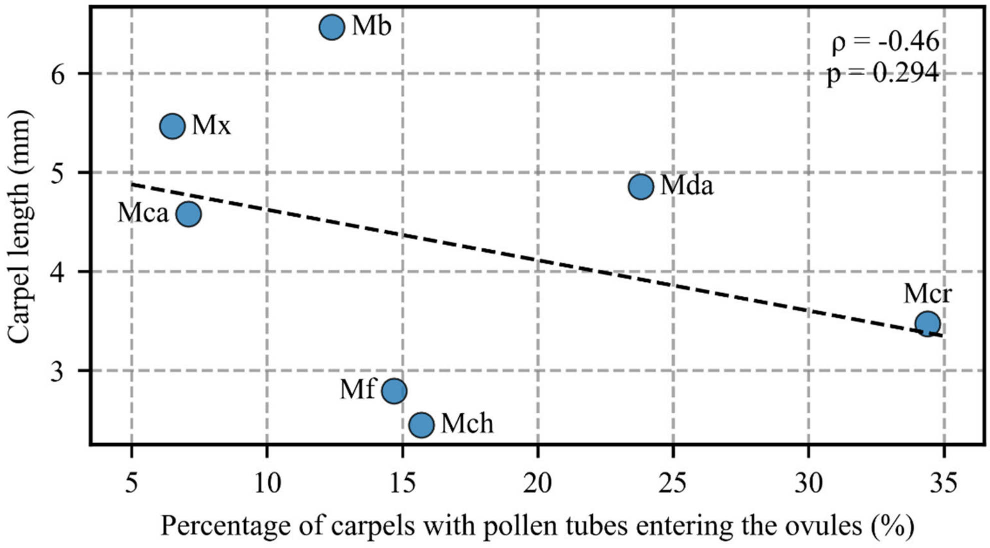

Figure S1. Correlation analysis between carpel length and ovule entry rate. The dashed line represents the linear regression trend. ρ indicates Spearman's rank correlation coefficient, and p represents the statistical significance level. Abbreviations: Mcr, *M. crassipes*; Mf, *M. figo*; Mca, *M. cavaleriei* var. *platypetala*; Mm, *M. macclurei*; Mb, *M. balansae*; Mch, *M. chapensis*; Mx, *M.* 'Xin'; Mda, *M.* 'Danxia'; Ml, *M. lotungensis*; Mde, *M. denudata*; Mg, *M. grandiflora*; Mi, *M. insignis*; Mco, *M. conifera*.
